# The Influence of Stretching the Hip Flexor Muscles on Performance Parameters. A Systematic Review with Meta-Analysis

**DOI:** 10.3390/ijerph18041936

**Published:** 2021-02-17

**Authors:** Andreas Konrad, Richard Močnik, Sylvia Titze, Masatoshi Nakamura, Markus Tilp

**Affiliations:** 1 Institute of Human Movement Science, Sport and Health, University of Graz, A-8010 Graz, Austria; richard.mocnik@uni-graz.at (R.M.); sylvia.titze@uni-graz.at (S.T.); markus.tilp@uni-graz.at (M.T.); 2 Institute for Human Movement and Medical Sciences, Niigata University of Health and Welfare, 1398 Shimami-cho, Kita-ku, Niigata 950-3198, Japan; masatoshi-nakamura@nuhw.ac.jp

**Keywords:** iliopsoas, rectus femoris, mobility, flexibility

## Abstract

The hip flexor muscles are major contributors to lumbar spine stability. Tight hip flexors can lead to pain in the lumbar spine, and hence to an impairment in performance. Moreover, sedentary behavior is a common problem and a major contributor to restricted hip extension flexibility. Stretching can be a tool to reduce muscle tightness and to overcome the aforementioned problems. Therefore, the purpose of this systematic review with meta-analysis was to determine the effects of a single hip flexor stretching exercise on performance parameters. The online search was performed in the following three databases: PubMed, Scopus, and Web of Science. Eight studies were included in this review with a total of 165 subjects (male: 111; female 54). In contrast to other muscle groups (e.g., plantar flexors), where 120 s of stretching likely decreases force production, it seems that isolated hip flexor stretching of up to 120 s has no effect or even a positive impact on performance-related parameters. A comparison of the effects on performance between the three defined stretch durations (30–90 s; 120 s; 270–480 s) revealed a significantly different change in performance (*p* = 0.02) between the studies with the lowest hip flexor stretch duration (30–90 s; weighted mean performance change: −0.12%; CI (95%): −0.49 to 0.41) and the studies with the highest hip flexor stretch duration (270–480 s; performance change: −3.59%; CI (95%): −5.92 to −2.04). Meta-analysis revealed a significant (but trivial) impairment in the highest hip flexor stretch duration of 270–480 s (SMD effect size = −0.19; CI (95%) −0.379 to 0.000; Z = −1.959; *p* = 0.05; I^2^ = 0.62%), but not in the lowest stretch duration (30–90 s). This indicates a dose-response relationship in the hip flexor muscles. Although the evidence is based on a small number of studies, this information will be of great importance for both athletes and coaches.

## 1. Introduction

Stretching is commonly used as a warm-up routine prior to physical activities, with the goal being to increase the range of motion (ROM) of a joint [1]. With regard to the impact on performance parameters (i.e., strength, speed) there is a debate as to whether stretching can be helpful as a warm-up. In their review, Behm et al. [2] reported mean performance impairments of 3.7% and 4.4% immediately after static stretching and proprioceptive neuromuscular facilitation (PNF) stretching, respectively, but an increase in performance of 1.3% after dynamic stretching. Both muscle tightness and muscle stiffness can be reduced by single stretching exercises [3,4]. However, whilst muscle tightness is defined as a limited range of motion, muscle stiffness is defined as the resistance to stretch [5].

The hip flexor muscles (e.g., musculus iliopsoas, rectus femoris) are major contributors to lumbar spine stability [6]. While a minimum amount of tightness is required for lumbar spine stability and health, hip flexors that are too tight pose a risk for lower back pain [7]. Hence, an optimum amount of ROM in the hip flexors is required. Hip flexors are defined as being tight if full hip extension in the end position of the modified Thomas test cannot be reached [8]. In addition to lower back pain, tight hip flexors can also compromise isometric trunk strength [9], which likely has detrimental effects on sports performance. Endo and Sakamoto [10] also reported relationships between tight hip flexors and reduced dynamic balance, as assessed by star excursion balance tests in the lateral direction. Hip flexor tightness can also lead to muscle fatigue and can negatively affect movement patterns [11,12]. Furthermore, reduced gluteus maximus activation and lower gluteus maximus to biceps femoris co-activation were reported in female soccer players with lower hip extension ROM, indicating adapted neuromuscular strategies that negatively influence movement patterns, and hence can lead to decreased performance and injury [13]. In summary, there is a body of evidence that hip flexors that are too tight likely have a negative effect on several performance parameters.

Sedentary behavior reduces hip extension flexibility and hence increases flexor tightness. An average of ≥8 h mean sedentary time has been reported in a youth population [14], and also in an elderly population [15]. It is therefore likely that most of the sedentary population have tight hip flexors. This is underlined by the findings of Mettler et al. [16], who reported that two-thirds of the investigated population had limited hip extension flexibility, and hence tight hip flexors.

Similar to the other muscles of the lower leg (i.e., plantar flexors [17]), hip flexor stretching decreases tightness (and hence increases hip extension ROM) following an acute bout of stretching [12]. This will likely counteract the aforementioned problems when applied repeatedly [16]. Thus, frequent hip flexor stretching could be a beneficial strategy to sustain or even increase hip extension ROM. This might also imply a reduction in lower back pain and the prevalence of injuries and likely lead to an increase in performance [18]. However, to date, no review has summarized the literature about the effects of a single hip flexor stretching exercise on sports performance.

Therefore, this systematic review with meta-analysis was aimed at identifying if a single bout of stretching of the hip flexors has an impact on performance parameters. Since isokinetic, balance, and sport-specific parameters describe the different skills and dimensions of performance, we divided the analyzed parameters into the following three groups. Group 1: isokinetic parameters (*peak torque, mean power output, work, joint angle at peak torque, acceleration*); Group 2: balance and proprioception parameters (*Y-balance test*, *joint position sense*); Group 3: sport-specific parameters (*sprint time, countermovement jump height, foot speed*).

## 2. Materials and Methods

### 2.1. Search Strategy and Risk of Bias Assessment

This review is based on the suggestions from Munn et al. [19] for systematic reviews with meta-analysis. This review considered studies where the participants stretched the hip flexor muscles exclusively. However, as the biarticular rectus femoris is responsible for hip flexion and knee extension, specific leg extensor stretches were also considered.

An electronic literature search was performed in the following three databases: PubMed, Scopus, and Web of Science. The search period ranged from 1990 until the end of May 2020. The keywords for the online search remained unchanged for all databases and were applied within the title and abstract. The detailed search strategy for each database is presented in Appendix A.

**Figure 1:**
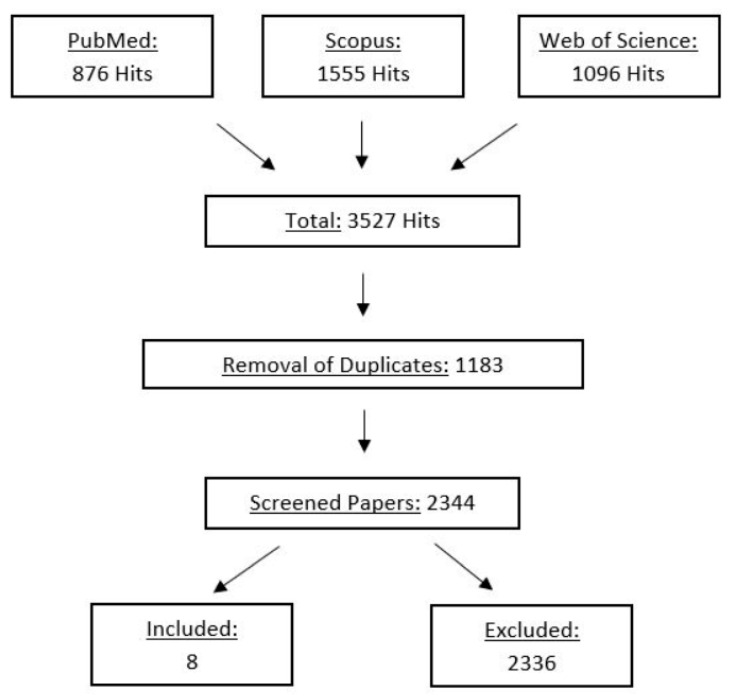
Flowchart of the systematic screening process (PRISMA).

A systematic search was done by two independent researchers (A.K., R.M.). In the first step, all hits were screened by their title. If the content of a study remained unclear, the abstract (and if necessary the full text) was screened to identify relevant papers. Following this independent screening process, the researchers compared their findings. Disagreements were resolved by jointly reassessing the studies against the eligibility criteria. Overall, 2344 papers were screened, from which finally eight papers were found to be eligible for this review. The full search process is illustrated as a flowchart in Figure 1. Additionally, a risk of bias assessment was conducted with the Cochrane risk of bias assessment tool [20]. Table 1 shows a high risk of bias in three out of the eight included studies. Two studies [21,22] had a high risk of selection bias because they followed an intervention protocol without a control group. Moreover, in one study, 10 out of 35 participants could not complete the whole study setup (4 visits) due to sore muscles from the previous visits [23]. Thus, a high risk of attrition bias has to be reported.

### 2.2. Inclusion/Exclusion Criteria

To be considered useful and therefore selected for this review, a study had to meet the following inclusion criteria: (1) the study was written in English and published after 1990 as an article in a peer-reviewed journal; (2) the study examined the effects of stretching (static, dynamic, PNF, or ballistic) on the hip flexor muscles exclusively; (3) the study was executed with healthy and pain-free individuals; (4) the results must include sport-specific performance parameters (e.g., peak torque, running speed, force production, balance, jump height, and others); and (5) the study had a pre/post stretching intervention design.

Studies were excluded if: (1) the intervention was performed on children or an elderly population; (2) muscles other than the hip flexors were stretched (except for leg extensors in combination with the rectus femoris); and (3) the study only focused on the effects of stretching on flexibility or ROM.

We then categorized the analyzed parameters into the following three groups. Group 1: isokinetic parameters (*peak torque, mean power output, work, joint angle at peak torque, acceleration* tested with an isokinetic dynamometer); Group 2: balance and proprioception parameters (*Y-balance test* [Y-Balance test kit], *joint position sense* [iPod touch device]); Group 3: sport-specific parameters (*sprint time* [electronic timing system], *countermovement jump height* [Vertec system], *foot speed* [high-frequency video camera]). We believed that this classification scheme would help to make the results more applicable, and would therefore allow a better understanding of which kind of sports/performance may be benefited by stretching the hip flexors. The applied tests are standard measures in sport science and showed (where assessable) a high reliability (e.g., isokinetic measures the ICC was >0.93 [21] or Vertex system for counter-movement jump assessment the ICC was 0.88 [25]).

### 2.3. Data Analysis

Percentage and/or absolute changes of the relevant parameters were extracted from the included studies. Mean values represent the means of the percentage changes weighted by the sample sizes of the respective studies. The 95% confidence interval (CI) and the median (since some data was not normally distributed) were calculated. Individual effect sizes were calculated when absolute mean values and standard deviations were reported based on the suggestions of Cohen [28]. The effect sizes 0.2, 0.5, and 0.8 were defined for a small, medium, and large effect, respectively [28].

Since the Shapiro-Wilk test was significant, a Kruskal-Wallis-test was used to determine the effect between the three stretching durations (defined in clusters. 30–90 s; 120 s; 270–480 s). If the Kruskal-Wallis-test was significant, a Mann-Whitney-U-test was used for pairwise comparisons between these groups. The meta-analysis was performed with the software Comprehensive Meta-Analysis according to the recommendations of Borenstein et al. [29]. Using a random-effects meta-analysis, we assessed the effect in terms of standardized mean difference (SMD). According to the recommendations of Hopkins et al. [30], we defined the effect for the SMD <0.2, 0.2–0.6, 0.6–1.2, 1.2–2.0, 2.0–4.0, >4.0 as trivial, small, moderate, large, very large, extremely large, respectively. I^2^ statistics were calculated to assess the heterogeneity among the included studies and thresholds of 25%, 50%, and 75% were defined to be a low, moderate, and high level of heterogeneity, respectively [31,32]. A meta-analysis was only conducted if a sufficient amount of studies (n ≥ 3) was involved in the analysis. An alpha level of 0.05 was defined for the statistical significance of all the tests.

## 3. Results

Eight studies of the acute effects of hip flexor stretching were included in this review. These studies included a total of 165 subjects (male: 111; female 54) and applied an average stretching duration of 242 ± 205 s (30 s to 480 s).

Table 2 reports detailed information about the population and the stretching exercises used in the included studies. Table 3 shows the outcomes for all the measured parameters.

### 3.1. Effect of Stretching Duration

**Figure 2:**
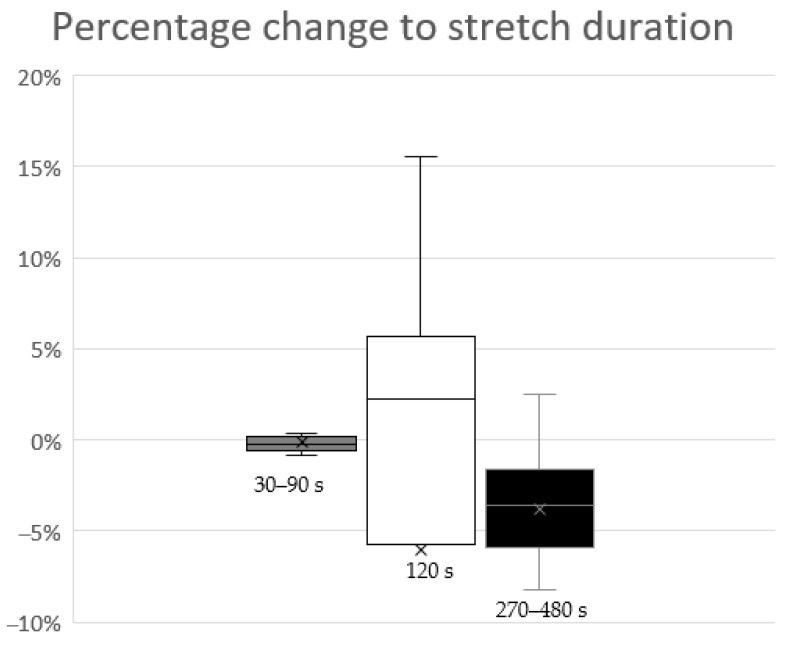
Boxplot diagram of the percentage change in performance parameters in relation to the stretching duration. The grey boxplot represents the parameter changes following a stretching duration of between 30 and 90 s. The white boxplot represents the parameter changes following a stretching duration of 120 s. The black boxplot represents the parameter changes following a stretching duration of between 270 and 480 s. Weighted means are represented by the “×” in or next to the box.

The mean, median, and confidence intervals of the weighted percentage changes in performance in the defined clusters of 30–90 s, 120 s, and 270–480 s were −0.12%; −0.28%; CI (95%): −0.49 to 0.41, −5.90%; 2.22%; CI (95%): −22.95 to 5.89, and −3.59%; −3.62% CI (95%): −5.92 to −2.04, respectively. The Kruskal-Wallis-test showed a significant effect between the three stretch durations (*p* = 0.006; H = 10.3). The post-hoc Mann-Whitney-U-test revealed a significant difference between the cluster 30–90 s and 270–480 s (*p* = 0.02), but no significant effect between the other groups. Within the cluster of 30–90 s, one parameter was significantly improved (sport-specific parameters), while the remaining eight were unchanged (5 isokinetic and 3 sport-specific parameters) (see also Table 4 for more information). Within the cluster of 120 s (only balance and proprioception parameters within one study; [12]), four parameters were significantly improved while the remaining six were unchanged. Within the cluster of 270–480 s, seven parameters were unchanged (1 sport-specific parameter and 6 isokinetic parameters), while the remaining 17 showed an impairment (isokinetic parameters only). Figure 2 shows boxplots of the percentage change in the performance parameters (including non-significant results), comparing pre and post values in relation to the stretching durations (30–90 s; 120 s; 270–480 s). In accordance, the meta-analysis showed no significant changes in performance in the cluster 30–90 s stretching, however, a significant decrease in performance in the cluster with a stretching duration between 270 and 480 s (trivial effect size) with all included parameters as well as for peak torque only (small effect size) (see Table 5). Note that 120 s stretching was applied in only one study and hence, no meta-analysis was performed. Moreover, due to technical reasons, the study of Young et al. [26] could not be implemented in the meta-analysis because the authors compared only between the intervention and control group but not within the intervention group.

### 3.2. Effect of Stretching Method

While 27 out of the 43 parameters were tested following a static stretching exercise (in seven studies), nine parameters were tested following PNF stretching (in two studies), and seven were tested following dynamic stretching (in two studies) (see Table 6 for details). With regard to static stretching, only a single measure of vertical jump performance (sport-specific parameter) was significantly improved [25], while 13 parameters did not change (11 isokinetic parameters and 2 sport-specific parameters), and 13 parameters (isokinetic parameters only) showed an impairment following the single static stretching exercise. Thus, in summary, the included studies (n = 7) which investigated the effects of static stretching on performance (isokinetic parameters and sport-specific parameters) revealed an average impairment of −2.54% (median: −1.56% CI (95%): −4.48 to −1.05) (see Table 6 for details). A meta-analysis with the static stretching studies showed no significant effect of a single static stretching exercise of the hip flexors on performance (effect size = −0.070; CI (95%) −0.202 to 0.061; Z = −1.048; *p* = 0.29; I^2^ = 27.75%). With regard to PNF stretching, two balance and proprioception parameters (*Y-balance test posteromedial* and *Y-balance test posterolateral*) showed a significant improvement, while three showed no change (*joint position sense at 30°*, *joint position sense at 60°*, and *Y-balance test anterior*) (all balance parameters out of one study), and four isokinetic parameters (*peak torque 60°/s* and *peak torque 300°/s*, *mean power 60°/s*, and *mean power 300°/s*) showed an impairment (all isokinetic measures out of one study). The included studies (n = 2) which investigated the effects of PNF stretching on performance (balance and proprioception parameters and isokinetic parameters) revealed an average impairment of −2.59% (median: −3.17% CI (95%): −9.54 to 3.63) (see Table 6 for details). Dynamic stretching led to a significant improvement in two parameters (*Y-balance test posteromedial* and *Y-balance test posterolateral*) and no significant change in the other five tested parameters (*40-yard sprint time*, *joint position sense at 30°*, *joint position sense* at *60°*, and *Y-balance test anterior*). The included studies (n = 2) which investigated the effects of dynamic stretching on performance (balance and proprioception parameters and sport-specific parameters) revealed an average impairment of −7.06% (median: 1.66% CI (95%): −30.45 to 4.36) (see Table 6 for details). Since only two studies investigated the effects of PNF stretching, dynamic stretching, respectively, no meta-analysis was performed in these groups.

### 3.3. Effects of Hip Flexor Stretching on Different Aspects/Dimensions of Performance

The eight included studies investigated 43 different performance-related parameters. Performance parameters were defined in this review as isokinetic parameters (e.g., *peak torque* and *mean power*), balance and proprioception parameters (e.g., *Y-balance test*), and sport-specific parameters (e.g., *sprint time*, *countermovement jump height*), but not flexibility (*ROM*). While five parameters showed a significant improvement following a hip flexor stretching exercise (4 balance and proprioception parameters and 1 sport-specific parameter), 21 showed no change (11 isokinetic parameters, 6 balance and proprioception parameters, and 4 sport-specific parameters), and 17 showed impairment in performance (isokinetic parameters only).

#### 3.3.1. Isokinetic Parameters

Four studies investigated the effects of a hip flexor stretching exercise on isokinetic strength parameters. The investigated parameters were *peak torque*, *mean power output*, *work*, *joint angle at peak torque*, and *acceleration*. All four studies reported either a significant decrease (17×) in the measured parameter or no change (11×). On average, the included studies revealed an impairment of −3.22% (median: −2.86%; CI (95%): −5.11 to −1.79). A meta-analysis revealed a significant trivial effect of a single stretching exercise on isokinetic performance parameters (effect size = –0.123; CI (95%) −0.209 to −0.037; Z = −2.812; *p* = 0.005; I^2^ = 0%).

#### 3.3.2. Sport-Specific Parameters

Three studies considered sport-specific parameters. These were *sprint time*, *countermovement jump height,* and *foot speed*. The studies showed either a significant improvement (1×) or no change (4×) in the measured parameters following a single stretching exercise of the hip flexors. The sport-specific parameters revealed an average improvement of 0.16% (median: 0.34%; CI (95%): −0.45 to 1.11). Since only two studies investigated the effects of stretching on sport-specific parameters (the study of Young et al. [26] could have not been included due to technical reasons) no meta-analysis was performed.

#### 3.3.3. Balance and Proprioception Parameters

Only one study tested the effects of a single stretching exercise of the hip flexors on balance and proprioception parameters. The investigated parameters were the *Y-balance test* and *joint position sense*. The study reported either a significant improvement (4×) or no change (6×) following a single stretching exercise of the hip flexors. These parameters revealed an average impairment of −5.90% (median: 2.22%; CI (95%): −22.95 to 5.89) Since only one study investigated the effects of stretching on balance and proprioception parameters, no meta-analysis was performed.

## 4. Discussion

### 4.1. Effect of Stretching Duration

With regard to static stretching, Behm and Chaouachi [33] showed in their review that static stretching (independent of which muscle) for more than 90 s has a high probability of decreasing force production and jump height. To rule out the likelihood of strength deficits, the authors suggested limiting static stretching exercises to 30 s or less for each muscle group prior to a task that requires “springiness”. A more recent review by Behm et al. [2] reported a greater loss in performance with static stretching of ≥60 s (−4.6%) compared to static stretching of <60 s (−1.1%). In addition, Kay and Blazevich [34] pointed out in their review that, in three-quarters of the involved studies, a static stretching exercise of less than 45 s did not affect muscle strength in terms of measured peak torque. In the studies considered in this review that included stretching for ≤90 s (n = 3), one parameter was significantly improved (sport-specific parameter), while the remaining eight parameters did not change (5 isokinetic parameters and 3 sport-specific parameters) (see also Table 4 for more detail). The average percentage change of all nine parameters with stretching durations for ≤90 s was −0.12% (median: −0.28%; CI (95%): −0.49 to 0.41). Additionally, the meta-analysis showed no significant effect in these nine parameters (see Table 5), which indicates that stretching the hip flexor for ≤90 s will result in neither an improvement nor an impairment in performance parameters. Only one study [12] investigated an intermediate stretching duration of 120 s and included the effects of both dynamic and PNF stretching exercises on balance and proprioception (see also Table 4 for more detail). In this review, out of the 10 considered measures, four showed a significant improvement *(Y-balance test posteromedial and Y-balance test posterolateral* in the dynamic stretching group, PNF stretching group, respectively), while the remaining six were unchanged *(joint position sense at 30°, joint position sense at 60°, and Y-balance test anterior* in the dynamic stretching group, PNF stretching group, respectively). However, on average, there was an average impairment of all 10 parameters (mean: −5.90%; median: 2.22%; CI (95%): −22.95 to 5.89). This can be explained by the high percentage changes (however not significant) since the baseline values of the *joint position sense* parameter were close to zero. Thus, minor absolute changes of this parameter led to high percentage changes. Hence, the averaged results of this review, with regard to balance and proprioception, and also PNF and dynamic stretching, should be viewed with caution. By excluding the *joint position sense* parameter, the average change due to hip flexor stretching on balance and proprioception would be an improvement of 3.44% (median: 2.22%; CI (95%): 1.54 to 3.95), instead of an impairment of −5.90% (median: 2.22%; CI (95%): −22.95 to 5.89). When longer stretching durations (270–480 s) were applied, seven out of the 24 parameters showed no significant change and 17 parameters showed a significant impairment, which is reflected in the average percentage change of −3.59 (median −3.62% CI (95%): −5.92 to −2.04) (see also Table 4 for details) and a significant effect in the meta-analysis (see also Table 5). However, the results of the meta-analysis revealed a trivial effect (SMD = −0.19) only, and hence, caution must be taken to not overemphasize this result. Zakas et al. [27] was the only study that compared the effect of a single short-duration static stretching exercise (60 s) to that of a longer-duration exercise (480 s). They reported no significant changes in quadriceps isokinetic peak torque following the short hip flexor stretching exercise, whereas they did find significant decreases in isokinetic peak torque following the longer-duration exercise. This result was confirmed by the comparison of the effects on performance between the three defined stretch durations (30–90s; 120 s; 270–480 s) in this review. The shortest stretch duration (30–90 s) was significantly different from the longest stretch duration (270–480 s) (see Figure 2). This finding, the findings from the meta-analysis (see Table 5), and the findings from Zakas et al. [27] suggest a similar dose-response relationship between stretching duration and performance for the hip flexor muscle and other lower leg muscles (e.g., plantar flexor muscles), as reported by several reviews [2,33,34]. A possible mechanism for such a dose-response relationship might be found in the decrease in muscle stiffness following stretching durations ≥120 s (e.g., [35,36]), whilst shorter durations (e.g., 60 s) did not lead to changes in muscle stiffness [37].

In summary, the existing data provide evidence that a single bout of hip flexor stretching of up to 120 s can have a positive effect on balance [12] and jump performance [25]. Moreover, up to a stretching duration of 120 s, no detrimental effect has been reported in the studies dealing with a sport-specific performance [23], balance [12], or isokinetic parameters [27], regardless of the stretching techniques used. In contrast to other muscle groups, where 120 s of stretching likely decreases force production (e.g., plantar flexors [38]), it seems likely that isolated hip flexor stretching with a moderate duration of up to 120 s has no detrimental effect or even a positive impact on performance-related parameters. This difference might at one hand be explained in the special characteristic of the hip flexor muscles. Compared to other lower limb muscles the hip flexor muscles, especially the iliopsoas, have a major function in lumbar spine stability [6] and hence, too-tight hip flexors can lead to a disadvantageous position of the pelvis. Consequently, this can cause muscle fatigue and can negatively affect movement patterns [11,12] and hence lead to major impairment in performance [9]. On the other hand, the tested movements that showed improvements (running, jumping) are rather characterized by hip extensions than hip flexing. Therefore, the hip flexors are not the main contributors but rather improve the movement conditions for the agonist muscles. It should be, however, noted that hip flexor stretching led to decreases, when tests directly measured hip flexion performance, e.g., peak torque. It can be therefore assumed that even longer stretching durations (≥60 s [2]) of the hip flexors, which are generally suggested to decrease performance do not necessarily lead to detrimental effects in movements where hip flexors are not the main movers.

Although the evidence is based on a small number of studies, this information will be of great importance for both athletes and coaches.

### 4.2. Effect of Stretching Method

All three investigated stretching techniques can lead to an impairment in performance following a single hip flexor stretching exercise. While dynamic stretching showed the greatest average impairment of all the parameters (−7.06% (median: 1.66% CI (95%): −30.45 to 4.36); average stretch duration: 94.2 s), PNF stretching and static stretching showed similar average impairments (PNF: −2.59% (median: −3.17% CI (95%): −9.54 to 3.63); average stretch duration: 280 s; static: −2.54% (median: −1.56% CI (95%): −4.48 to −1.05); average stretch duration: 363.3 s). At first glance, this goes against the findings of Behm et al. [2], who reported mean performance impairments of 3.7% and 4.4% immediately after static stretching and PNF stretching, respectively, but an increase in performance of 1.3% after dynamic stretching. However, removing the results of the *joint position sense* parameter, because of its high and misleading percentage change (since the values are close to zero), leads to more credible average changes of −2.54%, +1.64%, and −1.878% for static, dynamic, and PNF stretching, respectively. Thus, this small increase in performance following dynamic stretching and impairments following static and PNF stretching would underline the findings of Behm et al. [2].

Two studies included in this review compared the effects of different stretching methods. Wallmann et al. [23] compared the effects of single bouts of static, dynamic, or ballistic stretching of the hip flexors on the *40-yard sprint time* (sport-specific parameter). They found no significant difference between pre and post values within the two techniques, but a significant reduction in *sprint time* following a conventional warm-up without stretching. Aslan et al. [12] compared the effects of 120 s of dynamic and PNF stretching of the hip flexors on the *Y-balance test* (balance and proprioception parameter). Although both techniques were shown to be an effective way to improve balance parameters, the PNF technique provided greater positive effects than dynamic stretching [12]. This is in contrast to the findings of Behm et al. [2] on strength tasks, who reported an impairment of 4.4% following PNF stretching, but an increase in performance following a dynamic stretching protocol. Although strength and balance are related [39], an acute bout of stretching has different impacts on balance and strength parameters [40]. Thus, it can be assumed that the findings of Behm et al. [2] about strength tasks and the findings of Aslan et al. [12] about balance tasks are not totally comparable. Moreover, in the review of Behm et al. [2], the results were based on the stretching of several lower leg muscles, and this might not be valid for isolated stretching exercises for the hip flexor muscles, as presented in this review.

Most of the included studies (six out of eight) investigated the effects of a static stretching exercise of the hip flexors on performance and reported an average decrease of −2.54% (median: −1.56% CI (95%): −4.48 to −1.05 (see also Table 6 for more detail). However, no significant effect was shown in the meta-analysis (effect size = −0.070; CI (95%) −0.202 to 0.061; Z = −1.048; *p* = 0.29; I^2^ = 27.75%), indicating that this impairment was not significant. With regard to dynamic stretching the exclusion of the *joint position sense* parameter from the analysis in the study of Aslan et al. [12] changes the result substantially into an average improvement of 1.64%. However, since only five parameters (3 balance and proprioception parameters and 2 sport-specific parameters) out of two studies [12,23] were included in this analysis, these results should not be generalized and have to be interpreted with caution.

### 4.3. Effects of Hip Flexor Stretching on Different Aspects/Dimensions of Performance

#### 4.3.1. Isokinetic Parameters

The included studies which investigated the effects of a hip flexor stretching exercise on isokinetic strength parameters [21,22,24,27] reported either a decrease in the measured parameters or no change. This resulted in an average impairment of −3.22% (median: −2.86%; CI (95%): −5.11 to −1.79). Although the meta-analysis revealed that this change was significant, the magnitude showed a trivial effect only (effect size = −0.123; CI (95%) −0.209 to −0.037; Z = −2.812; *p* = 0.005; I^2^ = 0%). However, it should be mentioned that the studies which included isokinetic parameters mainly used stretching durations of 480 s [21,22,24], which was likely the underlying reason for the decrease in performance (see the review of Behm et al. [2]). This is supported by the study of Zakas et al. [27], who reported no change in quadriceps isokinetic performance following 60 s of static stretching, while 480 s of static stretching caused a decrease in performance. This has also been observed in similar studies of the plantar flexors, where static stretching of the calf muscles for 1 min [37] and 3 min [36] did not induce changes in maximum voluntary isometric contraction (MVC), while 5 min of static stretching caused a decrease [41]. 

#### 4.3.2. Sport-Specific Parameters

The sport-specific parameters such as *sprint time*, *countermovement jump height*, and *foot speed* investigated in three studies showed either an improvement or no change in performance, which resulted in an average improvement of 0.16% (median: 0.34%; CI (95%): −0.45 to 1.11). Since only five parameters were considered in the different stretching techniques (static (n = 3); dynamic (n = 2)), and durations ranging from 30 to 270 s were tested, no general conclusion should be made from this data. Wallmann et al. [23] compared the effects of single bouts of static, dynamic, or ballistic stretching of the hip flexors on the *40-yard sprint time*. They found no significant difference between pre and post values within the techniques, but a significant reduction in *sprint time* following a conventional warm-up without stretching. This result suggests that a warm-up including stretching of the hip flexors increases the chance of performance improvement. In addition, Behm et al. [2] concluded that post-stretching dynamic activities are able to counteract possible detrimental effects on performance following stretching, leading to a positive effect on performance. Thus, several authors have suggested including post-stretching dynamic activities in the warm-up regimes of athletes (see Behm et al. [2] for a review).

#### 4.3.3. Balance and Proprioception Parameters

The data of one study [12] showed that 120 s of hip flexor stretching can either improve or does not change balance and proprioception parameters. The average change of −5.90% (median: 2.22%; CI (95%): −22.95 to 5.89) indicates an overall impairment due to stretching. However, the results were substantially affected by the *joint position sense* parameter, due to its high percentage change (since the values were close to zero). Excluding this parameter changes the average impairment of −5.90% (median: 2.22%; CI (95%): −22.95 to 5.89) to an improvement of 3.44% (median: 2.22%; CI (95%): 1.54 to 3.95). Behm et al. [41] reported a decrease in balance and proprioception (compared to the control condition) following a 3 × 45 s static stretching exercise of the quadriceps, but also the hamstrings and plantar flexors. Since Aslan et al. [12] reported an improvement in some balance parameters following 120 s of stretching of the hip flexors, it can be speculated that stretching of the hip flexors has no adverse effect on balance. This is supported by Costa et al. [42], who found an improvement in the balance score following short-duration stretches (3 × 15 s), while the more prolonged stretching duration (3 × 45 s) did not cause balance performance changes.

A possible limitation of this review was that three out of the eight studies showed a high risk of bias (see Table 1 for details). Two studies [21,22] followed an intervention protocol without a control group which represents a high risk of selection bias. Moreover, in one study 10 out of 35 participants could not complete the whole study setup (4 visits) due to sore muscles from the previous visits [23]. Thus, a high risk of attrition bias has to be reported. However, these studies reported high reliability of their data, and hence, at least the measurement itself can be considered of high quality. Although significant effects were reported in some meta-analyses, the magnitudes of the effects were only trivial or small. Thus, caution must be taken not to overemphasize these results. However, we are confident that this systematic review with meta-analysis will be of great importance to get an overview on this topic and helps to develop further research hypotheses and projects.

## 5. Conclusions

The existing data provides evidence that a single bout of hip flexor stretching of up to 120 s can have a positive effect on balance (following dynamic stretching or PNF stretching) [12] and jump performance (following static stretching) [25]. Moreover, up to a stretching duration of 120 s, no detrimental effect has been reported in the studies dealing with sports-related performances [23], balance [12], or isokinetic parameters [27], regardless of the stretching techniques used. In contrast to other muscle groups such as the plantar flexors [38], where 120 s of stretching likely decreases force production, it seems likely that isolated hip flexor stretching of up to 120 s has no effect or even a positive impact on performance-related parameters. This difference might be explained by the specific function of the hip flexor muscles for lumbar spine stability. While too-tight hip flexors can lead to a disadvantageous position of the pelvis, stretching will lead to a more advantageous position of the lumbar spine and the pelvis. A comparison of the effects on performance between the three defined stretch durations (30–90 s; 120 s; 270–480 s) revealed a significantly different change in performance (*p* = 0.02) between the lowest hip flexor stretch duration (30–90 s; performance change: mean: −0.12%; median: −0.28%; CI (95%): −0.49 to 0.41) and the highest hip flexor stretch duration (270–480 s; performance change: mean −3.59%; median: −3.62% CI (95%): −5.92 to −2.04). Moreover, meta-analysis reported a significant impairment (but with a trivial effect size only) in the highest hip flexor stretch duration (270–480 s), whilst no significant effect was reported in the lowest hip flexor stretch duration (30–90 s) (see Table 5). This additionally indicates a dose-response relationship in the hip flexor muscles. Although the evidence is based on a small amount of studies, this information will be of great importance for both athletes and coaches. Based on our findings it can be recommended to stretch the hip flexor up to 120 s to improve performance, especially in sports where a high range of motion in the hip extension is required (e.g., dancing, gymnastics). Additionally, hip flexor stretching can be a preventive approach against injuries. Especially soccer players with tight hip flexors might benefit since tight hip flexors lead to greater activation of the synergistic rectus femoris which likely leads to overloads and/or fatigue of the muscle [43].

However, the limited amount of studies about the acute effects of PNF stretching (n = 2) and dynamic stretching (n = 2) on performance does not allow a clear conclusion to be made as to which stretching technique should be preferably applied to avoid performance impairment.

## 6. Perspective

While this review has shed some light on the effects of hip flexor stretching on performance, including some positive but also negative effects, more studies are needed to obtain a clearer picture of the effects of the various techniques and the dose-response relationship.

In addition to the immediate effect of a bout of stretching, it is also of great importance to understand the long-term effect of prolonged stretching training. Especially with regard to flexibility, it has been shown that repeated single stretches of a muscle-tendon unit can lead to enhanced flexibility in the long term. However, to date, only one study has investigated the effects of a hip flexion stretching intervention over 3 weeks on passive and sport-related flexibility and related kinematic changes of the lumbo-pelvic-hip complex during running [16]. The authors reported that an increase in passive hip extension flexibility cannot be transferred to an active movement during running. No studies are available that have investigated long-term hip flexor stretching training and its effects on lower back pain, the prevalence of injuries, and performance. Therefore, we strongly recommend long-term studies of hip flexor stretching in the future.

## Figures and Tables

**Table 1:** Risk of Bias Assessment with the Cochrane Tool.

	Random Sequence Generation (Selection Bias)	Allocation Concealment (Selection Bias)	Blinding of Participants and Personnel (Performance Bias)	Blinding of Outcome Assessment (Detection Bias)	Incomplete Outcome Data (Attrition Bias)	Selective Reporting (Reporting Bias)	Other Bias		
Aslan et al. [12]									Low risk of bias
Cramer et al. [21]									
Cramer et al. [22]									Unclear risk of bias
Marek et al. [24]									
Wakefield et al. [25]									High risk of bias
Wallmann et al. [23]									
Young et al. [26]									
Zakas et al. [27]									

**Table 2:** Summary of the Participants and Intervention Characteristics of the Studies which Investigated the Acute Effects of Hip Flexor Stretching on Performance.

Study	Population			Stretching Intervention			
	Subjects (m/f)	n	Age (Years)	Stretching Type	Stretching Method	Stretching Duration (Total Time)	Stretching Intensity
Aslan et al. [12]	m/f	36 (25/11)	22.37 ± 1.63	PNF (hold-relax)	6 × 20 s [10 s rest]	2 min/leg	<POD
				Dynamic	6 × 10 reps [10 s rest]	2 min/leg	<POD
Cramer et al. [21]	m/f	21 (7/14)	21.5 ± 1.3	Static	Unassisted: 4 × 30 s [20 s rest] + 3 × assisted: 4 × 30 s [20 s rest]	8 min/leg	POMD
Cramer et al. [22]	m/f	18 (8/10)	21.4 ± 3.0/23.0 ± 2.9	Static	Unassisted: 4 × 30 s [20 s rest] + 3 × assisted: 4 × 30 s [20 s rest]	8 min/leg	POMD
Marek et al. [24]	m/f	19 (9/10)	21 ±3/23 ± 3	Static	Unassisted: 4 × 30 s [20 s rest] + 3 × assisted: 4 × 30 s [20 s rest]	8 min/leg	POD
				PNF (contract-relax)	Unassisted: 4 × 30 s [20 s rest] + 3 × assisted: 4 × 30 s [20 s rest]	8 min/leg	POD
Wakefield et al. [25]	m	15	24.1 ± 2.4	Static	Assisted: 3 × 30 s [30 s rest]	90 s/leg	POMD
Wallmann et al. [23]	m/f	25 (16/9)	26.76 ± 2.42	Static	2 × 30 s	30 s/leg	<POD
				Dynamic	4 × 15 s	30 s/leg	<POD
				Ballistic	4 × 15 s	30 s/leg	<POD
Young et al. [26]	m	16	18–33	Static	2 × assisted: 6 × 30 s [30 s rest] + unassisted: 6 × 30 s [30 s rest]	4.5 min/leg	<POD
Zakas et al. [27]	m	15	25 ± 1.5	Static	Unassisted: 4 × 15 s [15 s rest]	1 min/leg	<POD
					Unassisted: 4 × 15 s [15 s rest] + assisted: 28 × 15 s [15 s rest]	8 min/leg	<POD

POD = point of discomfort, POMD = point of mild discomfort.

**Table 3:** Summary of the Results of the Studies which Investigated the Acute Effects of Stretching of the Hip Flexor Muscles.

Study	Stretching Type	Results (Performance)		Results (Range of Motion [°])	
		Outcome (Change in %)	Outcome (Δ-Values)	Hip	Knee
Aslan et al. [12]	PNF (hold-relax)	**JPS** 30°: ↑15.57% **JPS** 60°: ↓25.94% **Y-Test-A**: ↑1.38% **Y-Test-PM**: ↑2.78% * Y-Test-PL: ↑1.02% *	**JPS** 30°: ↑0.97 **PS** 60°: ↓0.55 **Y-Test-A**: ↑0.94 **Y-Test-PM**: ↑2.92 * Y-Test-PL: ↑1.15 *	**pROM**: ↑13.1 *	-
	Dynamic	**JPS** 30°: ↑7.01% **JPS** 60°: ↓72.82% **Y-Test-A**: ↑1.66% **Y-Test-PM**: ↑5.23% * Y-Test-PL: ↑3.65% *	**JPS** 30°: ↑0.42 **JPS** 60°: ↓0.75 **Y-Test-A**: ↑1.07 **Y-Test-PM**: ↑5.36 * Y-Test-PL: ↑3.97 *	**pROM**: ↑5.2 *	-
Cramer et al. [21]	Static	**PT** 60° s^−1^: ↓2.72% * PT 240° s^−1^: ↓4.18% * JAPT 60° s^−1^: ↓1.56% **JAPT** 240° s^−1^: ↑5.97% **MP** 60° s^−1^: ↓7.93% **MP** 240° s^−1^: ↑2.51%	**PT** 60° s^−1^: ↓5.5 * PT 240° s^−1^: ↓5.7 * JAPT 60° s^−1^: ↓1.0 **JAPT** 240° s^−1^: ↑3.1 **MP** 60° s^−1^: ↓10.5 **MP** 240° s^−1^: ↑5.9	-	-
Cramer et al. [22]	Static	**PT**: ↓3% * JAPT: no sign. change **MP**: no sign. change **Acc.**: ↓ 17.5% * Results presented as marginal means	nr	-	**pROM** 60° s^−1^: no sign. change; **pROM** 300° s^−1^: no sign. change
Marek et al. [24]	Static	**PT** 60° s^−1^: ↓0.16% **PT** 300° s^−1^: ↓1.68% **MP** 60° s^−1^: ↓0.37% **MP** 300° s^−1^: ↓2.62%	**PT** 60° s^−1^: ↓0.3 **PT** 300° s^−1^: ↓2.9 **MP** 60° s^−1^: ↓0.6 **MP** 300° s^−1^: ↓13.4	-	**aROM**: ↑1.8 * **pROM**: ↑1.8 *
	PNF (contract-relax)	**PT** 60° s^−1^: ↓5.96% * PT 300° s^−1^: ↓3.17% **MP** 60° s^−1^: ↓4.06% **MP** 300° s^−1^: ↓4.48%	**PT** 60° s^−1^: ↓10.9 **PT** 300° s^−1^: ↓3.7 **MP** 60° s^−1^: ↓6.6 **MP** 300° s^−1^: ↓22.9	-	**aROM**: ↑1.6 * pROM: ↑0.5 *
Wakefield et al. [25]	Static	**CMJ**: ↑1.74% *	**CMJ**: ↑1.02 *	**pROM**: ↑6.54% *	-
Wallmann et al. [23]	Static	**40-yard sprint time**: ↓0.17%	**40-yard sprint time**: ↓0.01	-	-
	Dynamic	**40-yard sprint time**: ↓0.87%	**40-yard sprint time**: ↓0.05		
	Ballistic	**40-yard sprint time**: ↑0.34%	**40-yard sprint time**: ↑0.02		
Young et al. [26]	Static	**Foot speed**: ↑0.49%	**Foot speed**: ↑0.1	**pROM**: ↑1.4	**pROM**: ↓1.7
Zakas et al. [27]	Static/1 min	**PT** 60° s^−1^: ↓0.28% **PT** 90° s^−1^: ↑0.05% **PT** 150° s^−1^: ↓0.46% **PT** 210° s^−1^: ↓0.61% **PT** 270° s^−1^: ↓0.56%	**PT** 60° s^−1^: ↓0.6 **PT** 90° s^−1^: ↑0.1 **PT** 150° s^−1^: ↓0.8 **PT** 210° s^−1^: ↓0.9 **PT** 270° s^−1^: ↓0.7	-	**pROM**: ↑4.1
	Static/8 min	**PT** 60° s^−1^: ↓5.54% * PT 90° s^−1^: ↓5.92% * PT 150° s^−1^: ↓7.22% * PT 210° s^−1^: ↓6.57% * PT 270° s^−1^: ↓8.19% *	**PT** 60° s^−1^: ↓11.7 * PT 90° s^−1^: ↓11.8 * PT 150° s^−1^: ↓12.7 * PT 210° s^−1^: ↓9.8 * PT 270° s^−1^: ↓10.4 *	-	**pROM**: ↑4.3

* = significant change; ↓ = performance decrease; ↑ = performance increase; Acc. = acceleration (ms); aROM = active range of motion (°); CMJ = countermovement jump height (cm); JAPT = joint angle at peak torque (°); JPS = joint position sense; MP = mean power output (W); OL-CMJ = one-leg countermovement jump height (cm); pROM = passive range of motion (°); PT = peak torque (Nm); VI = vascularity index (%); W = work (J); Y-Test-A = Y-balance test anterior (%); Y-Test-PL = Y-balance test posterolateral (%); Y-Test-PM = Y-balance test posteromedial (%); nr= not reported; Note that e.g., °/s = ° s^−1^.

**Table 4:** Summary of the Results of the Studies which Investigated the Acute Effects of Stretching of the Hip Flexor Muscles.

Study	Stretching Duration (s)	Stretching Type	Related Group of the Parameter	Change in %	Effect Size
Wallmann et al. [23]	30	Dynamic	sport-specific parameters	−0.87%	0.1
	30	Static	sport-specific parameters	−0.17%	0.02
	30	Ballistic	sport-specific parameters	0.34%	0.04
Zakas et al. [27] *	60	Static	isokinetic parameters	−0.28%	0.02
	60	Static	isokinetic parameters	0.05%	0.003
	60	Static	isokinetic parameters	−0.46%	0.03
	60	Static	isokinetic parameters	−0.61%	0.04
	60	Static	isokinetic parameters	−0.56%	0.04
Wakefield et al. [25]	90	Static	sport-specific parameters	1.74%	na
Aslan et al. [12]	120	Dynamic	balance and proprioception parameters	7.01%	0.14
	120	Dynamic	balance and proprioception parameters	−72.82%	0.28
	120	Dynamic	balance and proprioception parameters	1.66%	0.17
	120	Dynamic	balance and proprioception parameters	5.23%	0.61
	120	Dynamic	balance and proprioception parameters	3.65%	0.44
	120	PNF (hold-relax)	balance and proprioception parameters	15.57%	0.23
	120	PNF (hold-relax)	balance and proprioception parameters	−25.94%	0.15
	120	PNF (hold-relax)	balance and proprioception parameters	1.38%	0.17
	120	PNF (hold-relax)	balance and proprioception parameters	2.78%	0.25
	120	PNF (hold-relax)	balance and proprioception parameters	1.02%	0.11
Young et al. [26]	270	Static	sport-specific parameters	0.49%	0.12
Marek et al. [24]	480	PNF (contract-relax)	isokinetic parameters	−5.96%	0.18
	480	PNF (contract-relax)	isokinetic parameters	−3.17%	0.07
	480	PNF (contract-relax)	isokinetic parameters	−4.06%	0.12
	480	PNF (contract-relax)	isokinetic parameters	−4.48%	0.11
	480	Static	isokinetic parameters	−0.16%	0.005
	480	Static	isokinetic parameters	−1.68%	0.04
	480	Static	isokinetic parameters	−0.37%	0.01
	480	Static	isokinetic parameters	−2.62%	0.06
Zakas et al. [27] *	480	Static	isokinetic parameters	−5.54%	0.4
	480	Static	isokinetic parameters	−5.92%	0.41
	480	Static	isokinetic parameters	−7.22%	0.52
	480	Static	isokinetic parameters	−6.57%	0.46
	480	Static	isokinetic parameters	−8.19%	0.65
Cramer et al. [21]	480	Static	isokinetic parameters	−2.72%	0.11
	480	Static	isokinetic parameters	−4.18%	0.14
	480	Static	isokinetic parameters	−1.56%	0.2
	480	Static	isokinetic parameters	5.97%	0.29
	480	Static	isokinetic parameters	−7.93%	0.32
	480	Static	isokinetic parameters	2.51%	0.08
Cramer et al. [22]	480	Static	isokinetic parameters	Not reported	na
	480	Static	isokinetic parameters	Not reported	na
	480	Static	isokinetic parameters	−3.00%	na
	480	Static	isokinetic parameters	−17.50%	na

The results are sorted according to the stretch duration. Green color = significant improvement; Red color = significant impairment; Grey color = no significant Change; na = not available. * = Zakas et al. [27] had two stretch durations (60 s and 480 s).

**Table 5:** Meta-Analysis of the Effects of the Different Stretching Durations (Clusters 30–90 s and 270–480 s) on Performance Parameters. SDM = Standardized Difference in Means; * = Significant Effect for SDM.

			Effect Size				Heterogeneity
Stretching Duration	N Studies	N Measures	SDM	95% CI	Z	*p*	I^2^
30–90 s	3	9	0.135	[−0.168 to 0.438]	0.874	0.382	11.95%
270–480 s	4	24	−0.19	[−0.379 to 0.000]	−1.959	0.05 *	0.62%
270–480 s (only peak torque)	4	12	−0.206	[−0.385 to −0.027]	−2.257	0.02 *	0.97%

**Table 6:** Summary of the Results of the Studies which Investigated the Acute Effects of Stretching of the Hip Flexor Muscles.

Study	Stretching Duration (s)	Stretching Type	Related Group of the Parameter	Change in %	Effect Size
Wallmann et al. [23]	30	Ballistic	sport-specific parameters	0.34%	0.04
	30	Dynamic	sport-specific parameters	−0.87%	0.1
Aslan et al. [12]	120	Dynamic	balance and proprioception parameters	7.01%	0.14
	120	Dynamic	balance and proprioception parameters	−72.82%	0.28
	120	Dynamic	balance and proprioception parameters	1.66%	0.17
	120	Dynamic	balance and proprioception parameters	5.23%	0.61
	120	Dynamic	balance and proprioception parameters	3.65%	0.44
	120	PNF (hold-relax)	balance and proprioception parameters	15.57%	0.23
	120	PNF (hold-relax)	balance and proprioception parameters	−25.94%	0.15
	120	PNF (hold-relax)	balance and proprioception parameters	1.38%	0.17
	120	PNF (hold-relax)	balance and proprioception parameters	2.78%	0.25
	120	PNF (hold-relax)	balance and proprioception parameters	1.02%	0.11
Marek et al. [24]	480	PNF (contract-relax)	isokinetic parameters	−5.96%	0.18
	480	PNF (contract-relax)	isokinetic parameters	−3.17%	0.07
	480	PNF (contract-relax)	isokinetic parameters	−4.06%	0.12
	480	PNF (contract-relax)	isokinetic parameters	−4.48%	0.11
Wallmann et al. [23]	30	Static	sport-specific parameters	−0.17%	0.02
Zakas et al. [27]	60	Static	isokinetic parameters	−0.28%	0.02
	60	Static	isokinetic parameters	0.05%	0.003
	60	Static	isokinetic parameters	−0.46%	0.03
	60	Static	isokinetic parameters	−0.61%	0.04
	60	Static	isokinetic parameters	−0.56%	0.04
Wakefield et al. [25]	90	Static	sport-specific parameters	1.74%	na
Young et al. [26]	270	Static	sport-specific parameters	0.49%	0.12
Marek et al. [24]	480	Static	isokinetic parameters	−0.16%	0.005
	480	Static	isokinetic parameters	−1.68%	0.04
	480	Static	isokinetic parameters	−0.37%	0.01
	480	Static	isokinetic parameters	−2.62%	0.06
Zakas et al. [27]	480	Static	isokinetic parameters	−5.54%	0.4
	480	Static	isokinetic parameters	−5.92%	0.41
	480	Static	isokinetic parameters	−7.22%	0.52
	480	Static	isokinetic parameters	−6.57%	0.46
	480	Static	isokinetic parameters	−8.19%	0.65
Cramer et al. [21]	480	Static	isokinetic parameters	−2.72%	0.11
	480	Static	isokinetic parameters	−4.18%	0.14
	480	Static	isokinetic parameters	−1.56%	0.2
	480	Static	isokinetic parameters	5.97%	0.29
	480	Static	isokinetic parameters	−7.93%	0.32
	480	Static	isokinetic parameters	2.51%	0.08
Cramer et al. [22]	480	Static	isokinetic parameters	Not reported	na
	480	Static	isokinetic parameters	Not reported	na
	480	Static	isokinetic parameters	−3.00%	na
	480	Static	isokinetic parameters	−17.50%	na

The results are sorted according to the stretching technique. Green color = significant improvement; Red color = significant impairment; Grey color = no significant change; na = not available.

## Data Availability

All data generated or analyzed during this study are included in this published article.

## Appendix A

*PubMed:*

(((stretch * [Title/Abstract] OR mobility [Title/Abstract] OR flexibility [Title/Abstract] OR “range of motion” [Title/Abstract] OR rom [Title/Abstract]) AND (“hip joint” [Title/Abstract] OR pelvis [Title/Abstract] OR iliacus [Title/Abstract] OR “psoas major” [Title/Abstract] OR “rectus femoris” [Title/Abstract] OR iliopsoas [Title/Abstract] OR “hip flexor *” [Title/Abstract] OR “hip extension” [Title/Abstract])) AND (sport * [Title/Abstract] OR performance [Title/Abstract] OR jump * [Title/Abstract] OR run * [Title/Abstract] OR swim * [Title/Abstract] OR cycl * [Title/Abstract] OR bike * [Title/Abstract] OR strength [Title/Abstract] OR activation [Title/Abstract] OR sprint * [Title/Abstract] OR economy [Title/Abstract] OR mvc [Title/Abstract] OR “maximum voluntary contraction” [Title/Abstract] OR power [Title/Abstract] OR “stride length” [Title/Abstract] OR force [Title/Abstract] OR speed [Title/Abstract])) NOT (disease [Title/Abstract] OR palsy [Title/Abstract] OR syndrome [Title/Abstract] OR elderly [Title/Abstract] OR “back pain” [Title/Abstract]).

*Scopus:*

TITLE-ABS-KEY (*stretch **) OR TITLE-ABS-KEY (*mobility*) OR TITLE-ABS-KEY (*flexibility*) OR TITLE-ABS-KEY (*“range of motion”*) OR TITLE-ABS-KEY (*rom*) AND TITLE-ABS-KEY (*“hip joint”*) OR TITLE-ABS-KEY (*pelvis*) OR TITLE-ABS-KEY (*iliacus*) OR TITLE-ABS-KEY (*“psoas major”*) OR TITLE-ABS-KEY (*“rectus femoris”*) OR TITLE-ABS-KEY (*iliopsoas*) OR TITLE-ABS-KEY (*“hip flexor *”*) OR TITLE-ABS-KEY (*“hip extension”*) **AND** TITLE-ABS-KEY (*sport **) OR TITLE-ABS-KEY (*performance*) OR TITLE-ABS-KEY (*jump **) OR TITLE-ABS-KEY (*run **) OR TITLE-ABS-KEY (*swim **) OR TITLE-ABS-KEY (*cycl **) OR TITLE-ABS-KEY (*bike **) OR TITLE-ABS-KEY (*strength*) OR TITLE-ABS-KEY (*activation*) OR TITLE-ABS-KEY (*sprint **) OR TITLE-ABS-KEY (*economy*) OR TITLE-ABS-KEY (*mvc*) OR TITLE-ABS-KEY (*“maximum voluntary contraction”*) OR TITLE-ABS-KEY (*power*) OR TITLE-ABS-KEY (*“stride length”*) OR TITLE-ABS-KEY (*force*) OR TITLE-ABS-KEY (*speed*) **AND NOT** TITLE-ABS-KEY (*disease*) OR TITLE-ABS-KEY (*palsy*) OR TITLE-ABS-KEY (*syndrome*) OR TITLE-ABS-KEY (*elderly*) OR TITLE-ABS-KEY (*“back pain”*) AND (EXCLUDE (SUBJAREA, *“ENGI”*) OR EXCLUDE (SUBJAREA, *“COMP”*) OR EXCLUDE (SUBJAREA, *“AGRI”*) OR EXCLUDE (SUBJAREA, *“MATE”*) OR EXCLUDE (SUBJAREA, *“CENG”*) OR EXCLUDE (SUBJAREA, *“SOCI”*) OR EXCLUDE (SUBJAREA, *“MATH”*) OR EXCLUDE (SUBJAREA, *“VETE”*) OR EXCLUDE (SUBJAREA, *“PHYS”*) OR EXCLUDE (SUBJAREA, *“ENVI”*) OR EXCLUDE (SUBJAREA, *“IMMU”*) OR EXCLUDE (SUBJAREA, *“ARTS”*) OR EXCLUDE (SUBJAREA, *“PHAR”*) OR EXCLUDE (SUBJAREA, *“CHEM”*) OR EXCLUDE (SUBJAREA, *“EART”*) OR EXCLUDE (SUBJAREA, *“ENER”*) OR EXCLUDE (SUBJAREA, *“DENT”*) OR EXCLUDE (SUBJAREA, *“BUSI”*) OR EXCLUDE (SUBJAREA, *“DECI”*) OR EXCLUDE (SUBJAREA, *“BIOC”*)) AND (LIMIT-TO (PUBYEAR, *2020*) OR LIMIT-TO (PUBYEAR, *2019*) OR LIMIT-TO (PUBYEAR, *2018*) OR LIMIT-TO (PUBYEAR, *2017*) OR LIMIT-TO (PUBYEAR, *2016*) OR LIMIT-TO (PUBYEAR, *2015*) OR LIMIT-TO (PUBYEAR, *2014*) OR LIMIT-TO (PUBYEAR, *2013*) OR LIMIT-TO (PUBYEAR, *2012*) OR LIMIT-TO (PUBYEAR, *2011*) OR LIMIT-TO (PUBYEAR, *2010*) OR LIMIT-TO (PUBYEAR, *2009*) OR LIMIT-TO (PUBYEAR, *2008*) OR LIMIT-TO (PUBYEAR, *2007*) OR LIMIT-TO (PUBYEAR, *2006*) OR LIMIT-TO (PUBYEAR, *2005*) OR LIMIT-TO (PUBYEAR, *2004*) OR LIMIT-TO (PUBYEAR, *2003*) OR LIMIT-TO (PUBYEAR, *2002*) OR LIMIT-TO (PUBYEAR, *2001*) OR LIMIT-TO (PUBYEAR, *2000*) OR LIMIT-TO (PUBYEAR, *1999*) OR LIMIT-TO (PUBYEAR, *1998*) OR LIMIT-TO (PUBYEAR, *1997*) OR LIMIT-TO (PUBYEAR, *1996*) OR LIMIT-TO (PUBYEAR, *1995*) OR LIMIT-TO (PUBYEAR, *1994*) OR LIMIT-TO (PUBYEAR, *1993*) OR LIMIT-TO (PUBYEAR, *1992*) OR LIMIT-TO (PUBYEAR, *1991*) OR LIMIT-TO (PUBYEAR, *1990*)) AND (LIMIT-TO (LANGUAGE, *“English”*)).

*Web of Science:*

TS = (stretch * OR mobility OR flexibility OR “range of motion” OR rom) AND TS = (“hip joint” OR pelvis OR iliacus OR “psoas major” OR “rectus femoris” OR iliopsoas OR “hip flexor*” OR “hip extension”) AND TS = (sport * OR performance OR jump * OR run * OR swim * OR cycl * OR bike * OR strength OR activation OR sprint * OR economy OR mvc OR “maximum voluntary contraction” OR power OR “stride length” or force OR speed) NOT TS = (disease OR palsy OR syndrome OR elderly OR “back pain”).
